# A Single-Center Retrospective Study of Bacterial Infections After Acute Ischemic Stroke: The Prevalence Before and During the COVID-19 Pandemic

**DOI:** 10.3390/medicina60111755

**Published:** 2024-10-25

**Authors:** Weny Rinawati, Abdulloh Machin, Aryati Aryati

**Affiliations:** 1 Doctoral Program of Medical Science, Faculty of Medicine, Universitas Airlangga, Surabaya 60132, Indonesia; weny.rinawati-2022@fk.unair.ac.id; 2 Department of Clinical Pathology, Laboratory and Blood Bank, National Brain Center Hospital Prof. Dr. dr. Mahar Mardjono, Jakarta 13630, Indonesia; 3 Department of Neurology, Faculty of Medicine, Universitas Airlangga, Surabaya 60132, Indonesia; 4 Airlangga University Hospital, Surabaya 60132, Indonesia; 5 Dr. Soetomo General Academic Hospital, Surabaya 60132, Indonesia; 6 Department of Clinical Pathology, Faculty of Medicine, Universitas Airlangga, Surabaya 60132, Indonesia

**Keywords:** bacterial infection, COVID-19, culture, ischemic stroke, prevalence

## Abstract

*Background and Objectives*: The management of ischemic stroke involves care that is integrated and comprehensive, including the prevention of infection complications. This study aimed to investigate the prevalence and profile of bacterial infections after acute ischemic stroke both before and during the coronavirus disease 2019 (COVID-19) pandemic. *Materials and Methods*: A retrospective cross-sectional study examined the medical records of hospitalized acute ischemic stroke patients who had microbiological cultures taken at the National Brain Center Hospital Prof. Dr. dr. Mahar Mardjono, Jakarta, Indonesia, from 1 January 2018 to 31 December 2021. The percentage of positive bacterial growth in the microbiological cultures was used to determine the prevalence of bacterial infection after acute ischemic stroke. *Results*: A total of 519 patients met the study criteria, including 48 and 471 patients with infections before and during the COVID-19 pandemic. The prevalence of bacterial infection after acute ischemic stroke was 17.9%. There were significant differences in the prevalence of bacterial infection after acute ischemic stroke before and during the COVID-19 pandemic (87.5% vs. 10.8%). *Staphylococcus* sp. and *Klebsiella* sp. were the most frequently observed. The risk factors that influenced bacterial infection after acute ischemic stroke were intensive care stay (OR 0.22; 95%CI 0.13–0.39, *p*-value < 0.001), sepsis (OR 1.99; 95%CI 1.12–3.53, *p*-value = 0.019), COVID-19 infection (OR 4.81; 95%CI 2.64–8.76, *p*-value < 0.001), the use of steroids (OR 0.31; 95%CI 0.14–0.67, *p*-value = 0.003), and the use of TPN (OR 0.34; 95%CI 0.13–0.86, *p*-value = 0.022). *Conclusions*: Following the start of the COVID-19 pandemic, there was a decrease in the prevalence of bacterial infections after AIS. Patients with bacterial infections had different profiles before and during the COVID-19 pandemic.

## 1. Introduction

An acute ischemic stroke is the result of an abrupt arterial blockage brought on by thrombosis or an embolism within a week after the onset of a stroke [1]. Care for ischemic stroke should be integrated, complete, and comprehensive, including the prevention of complications that can affect stroke outcomes, as stroke has been one of the major causes of illness morbidity, mortality, and economic burden globally in the last 20 years [2,3]. During the COVID-19 pandemic, there was concern about increased morbidity and mortality due to failure to seek timely treatment [4,5,6]. The incidence varied widely between studies, and the outcomes of acute ischemic stroke patients with COVID-19 have been discussed elsewhere. In the meantime, there were patients with acute ischemic stroke (AIS) receiving treatment during the COVID-19 pandemic who also encountered complications, including infection after stroke [7].

Most infections after stroke occur in the first three to seven days in stroke patients who experience complications during acute care. The most frequently encountered infections are lower respiratory tract infections (RTIs) [7,8]. Sepsis may develop seven days after stroke onset, of which most infections are pneumonia or lung-related infections [9]. Although the last study showed that the COVID-19 pandemic did not increase the occurrence of infection after stroke, patient characteristics determine the risk factor for infection [6].

Prior research showed that anatomical, clinical, and iatrogenic factors could influence and predict infection after stroke [10,11,12]. According to earlier research, anatomical factors expected to influence infection after AIS were infarct location and volume. Infarct volume could determine the extent of lymphocytopenia and monocyte dysfunction, a main predictor for subsequent infections [13]. Age and leukocyte count, on the other hand, might predict pneumonia after stroke with 77.6% sensitivity and 84.0% specificity. Dysphagia could predict pneumonia after stroke with 76.0% sensitivity and 88.0% specificity [10]. In addition, a prior meta-analysis on risk factors for post-stroke pneumonia showed that besides age, male sex, stroke severity, dysphagia, nasogastric tube use, diabetes, ventilator use, smoking, chronic obstructive pulmonary disease, and atrial fibrillation were predictors [14].

However, most studies of infection after AIS did not focus on infection diagnosis based on the gold standard for diagnosing infectious diseases. Even though the main methods for diagnosing infection nowadays are represented by a few cutting-edge laboratory technologies, the gold standards in microbiological culture for infection diagnosis have historically been microbiological techniques like staining and culture [15].

Therefore, the aim of this study was to evaluate the prevalence and profile of bacterial infection after AIS based on microbiological culture before and during the COVID-19 pandemic using medical records from a neurological center. The data collected were analyzed to examine the effect of some factors on the risk of infection after AIS, and we hypothesized that there are differences in risk factors for infection after AIS before and during the COVID-19 pandemic.

## 2. Materials and Methods

### 2.1. Study Design and Participants

This study is retrospective with a cross-sectional design. This study, being non-interventional, was approved by the Research Ethics Committee, and the institutional review board waived the need for informed consent. We identified and reviewed the medical records and microbiological cultures of adult patients with a diagnosis of acute ischemic stroke who were hospitalized at the National Brain Center Hospital (NBC) Prof. Dr. dr. Mahar Mardjono, Jakarta, Indonesia, from 1 January 2018 to 31 December 2021. The NBC Hospital is a regional tertiary neurology teaching and referral hospital that offers a wide range of neurological health services, including stroke care.

The inclusion criteria for this study were patients aged 18 years or older, diagnosed with AIS within seven days of stroke onset, and who underwent microbiological culture. A microbiological culture was performed based on the attending physician’s clinical decision. As we were interested in comparing bacterial infections after AIS, we excluded patients with microbiologically proven fungal results from cultures. This study included all complete medical records of hospitalized patients who met the inclusion criteria.

The medical records provided demographic data, possible risk factors based on prior research, and other examination data. The data extracted from medical records were kept anonymous. Of the various risk factors for infection after AIS, this study accommodated some factors found in previous studies. From anatomical factors, this study included the number of infarct locations. In terms of clinical factors, this study included age, sex, comorbidity (such as diabetes mellitus (DM), human immunodeficiency virus (HIV) infection, and COVID-19 infection), leukocyte count results, treatment history (thrombolytic, thrombectomy, antibiotic, steroid, total parenteral nutrition (TPN), and transfusion), and procedure history (tracheostomy, digital subtraction angiography (DSA), and head surgery). The iatrogenic factors included were the use of medical devices, ventilators, a central venous catheter (CVC), a nasogastric tube (NGT), and a urinary catheter.

We compared patients admitted with acute ischemic stroke before the COVID-19 pandemic (2018–2019) and during the COVID-19 pandemic (2020–2021).

### 2.2. Definitions

An acute ischemic stroke is the result of an abrupt arterial blockage brought on by thrombosis or an embolism within a week after the onset of a stroke [1]. Infection after AIS was defined as any infection occurring in the acute phase of ischemic stroke [16,17], whereas sepsis was defined based on Sepsis-3 criteria or a result of the procalcitonin test > 0.5 ng/mL [18]. Procalcitonin was quantified via a blood specimen in the Cobas^®^ c501 clinical chemistry analyzer (Roche Diagnostics GmBH, Mannheim, Germany) using the chemiluminescent assay sandwich principle (Elecsys BRAHMS PCT, Cobas^®^, Roche Diagnostics GmBH, Mannheim) [19].

Infarct location was expertized by radiologists and categorized as single if there was only one location and multiple if there was more than one location based on the location in relation to the brain hemisphere.

Fasting blood glucose was examined using a blood specimen. The level of more than 126 mg/dL (7.0 mm/L) was defined as type 2 DM [20]. To measure blood glucose, Cobas^®^ c501 clinical chemistry analyzer application (Roche Diagnostics GmBH, Mannheim) with the sandwich principle of the chemiluminescent test (GLUC3, Cobas^®^, Roche Diagnostics GmBH, Mannheim) was used [21]. An HIV-infected patient was defined based on the reactivity of three consecutive reactive HIV rapid diagnostic tests according to the WHO’s proposal [22]. INDEC^®^ HIV ½ & Syphilis Combo (Indec Diagnostics, Jakarta, Indonesia) [23], StandardTM Q HIV ½ Ab 3-Line (SD Biosensor, Chungcheongbuk-do, Republic of Korea) [24], and Rapidan^®^ Testes Anti-HIV ½ Test (TurkLab, Izmir, Turkey) [25] were used as assay-1, assay-2, and assay-3, respectively. Confirmed COVID-19 infection was based on a polymerase chain reaction assay (Real-Q 2019-nCoV detection kit, BioSewoom, Seoul, Republic of Korea) for Severe Acute Respiratory Syndrome Coronavirus 2 [26,27].

Leukocyte count was examined using an ethylenediaminetetraacetic acid (EDTA) blood specimen in an automated hematology analyzer, Sysmex XN-1000 (Sysmex Indonesia, Jakarta, Indonesia). The reference range for leukocyte count assigned leukocyte counts more than 10,000/μL and less than 5000/μL, respectively, as leukocytosis and leukopenia [28].

Microbiological culture is a method of multiplying microbial organisms from infected tissue or body fluid specimens using the Vitek 2 Compact platform (BioMerieux, Lyon, France), allowing for identifications of the etiologic agents [7,29]. Specimen collection from sterile (such as cerebrospinal fluid (CSF) or blood) and non-sterile sites (such as respiratory tract specimens, pleural fluid, ulcers, or urine) proceeded with microbiological culture, which used previously described methods to determine the microorganisms [30].

The cerebrospinal fluid sediment resulting from the centrifugation of CSF specimens was directly examined under a microscope to confirm suspicious encapsulated microorganisms before being cultured on sheep blood agar. The blood specimen was inoculated into a BacT/ALERT^®^ bottle using a BacT/ALERT^®^ 3D 60 instrument (BioMerieux, Lyon, France) before performing Gram staining and sub-culture on sheep blood agar [30].

Bronchial lavage or sputum could be used as respiratory tract specimens. Gram staining was performed on a portion of the specimens, and the remainder were cultured on sheep blood agar. To isolate colonies for identification and susceptibility testing, as well as to measure colony-forming units per milliliter (CFU/mL), the urine specimens were processed using calibrated loops for plating before being cultured on sheep blood agar [30].

Colony morphology on the agar was observed after the incubation of sheep blood agar. Gram staining was used to identify the colonies chosen from the plate. A homogenous organism suspension was prepared by adding several morphologically similar colonies to sterile saline with a density equivalent to McFarland No. 1.80 to 2.20 using a calibrated VITEK^®^ 2 DensiCHEK™ Plus instrument. The suspension was processed using the VITEK system (bioMérieux, Lyon, France) [29,30].

Positive bacterial culture was defined as bacterial growth in the specimen culture; otherwise, it was negative. The prevalence of bacterial infection after AIS was based on the percentage of positive bacterial growth in microbiological cultures divided by the total number of subjects tested. According to the year of admission based on the COVID-19 pandemic, we defined two groups as before (2018–2019) and during (2020–2021) the pandemic.

### 2.3. Data Analysis

The characteristics of the research subjects are presented descriptively. The normality test of data distribution was performed using the Kolmogorov–Smirnov test. Non-normally distributed data are presented in the form of interquartile ranges (IQRs) and medians in descriptive statistics for continuous data, whereas number (*n*) and percentage (%) were used for categorical variables. Characteristic differences between the two categorical variables of before and during the pandemic were compared using Chi-square analysis or Fisher’s exact test. *p*-values were calculated from 2-sided tests.

Risk factors that influence infection after AIS were obtained and analyzed using the multivariate logistic regression analysis. In a univariate analysis, *p*-values less than 0.25 were accepted for inclusion in the multivariate logistic regression analysis to discover the characteristics linked with the positive rate of bacterial growth. To ensure the simultaneous consideration of multiple confounding variables, the multivariate logistic regression model was utilized. Both main and confounding variables were included throughout the whole modeling process, and interaction variables were eliminated one at a time based on how important they were to the study. Confounding was suggested if there was a change in the main variable’s odds ratio (OR) of greater than 10%. *p*-values less than 0.05 were considered statistically significant. The Statistical Package for the Social Sciences (IBM^®^ SPSS^®^ Statistics) version 26 was used for the analyses.

## 3. Results

**Figure 1:**
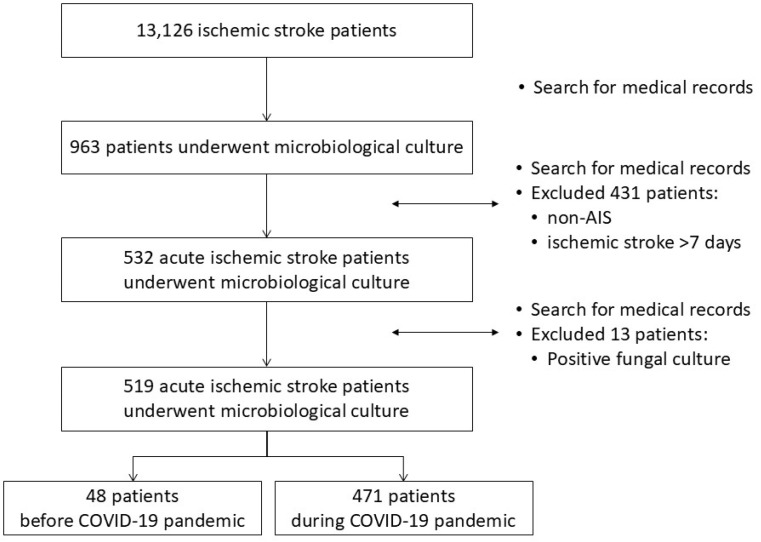
Flowchart of study participants.

There were 13,126 ischemic stroke patients based on the medical records of inpatients from 1 January 2018 to 31 December 2021. Specimen cultures were performed for 963 patients, and among them, we excluded 431 patients with non-AIS who had ischemic stroke more than seven days ago and 13 patients with a positive fungal culture. A total of 519 patients who met the inclusion criteria were enrolled, including 48 in the 2018–2019 group and 471 in the 2020–2021 group (Figure 1).

The mean age was 60 (10.5) years old. There were more male patients, 347 (66.9%), than female patients (172 (33.1%)). Pneumonia was the largest proportion of primary diagnosis and manifestation of infection (230, 44.3%). The most common specimen of the culture was blood (401, 77.3%), and sepsis occurred in 191 (36.8%) patients. Details of the patients’ characteristics before and during the COVID-19 pandemic are provided in Table 1.

Only 31.4% of patients were in intensive care, with the highest use of medical equipment and treatment being CVC, at more than 20%, and antibiotics, at around 70%. Nearly half of the clinical manifestations were pneumonia, which was the most common. The most prevalent comorbidity was COVID-19, followed by DM. From the leukocyte count, there were more cases of leukocytosis compared to the others. More than 50 percent of patients had a history of single-infarction lesions, and the most prevailing procedure was tracheostomy, even though not many procedures were performed on the patients.

### 3.1. Subjects’ Characteristics Between Groups

There were no differences in age and sex. Most during-pandemic patients were in non-intensive care units (35.4 vs. 72.0%, *p*-value < 0.001). There were no differences in the use of CVCs, NGTs, urinary catheters, transfusion, tracheostomy, or head surgery. However, during-pandemic patients had less use of ventilators (22.9 vs. 8.7%, *p*-value = 0.005), antibiotics (83.3 vs. 63.9%, *p*-value = 0.007), steroids (35.4 vs. 7.2%, *p*-value < 0.001), and TPN (22.9 vs. 4.7%, *p*-value < 0.001). Compared with the before-pandemic patients, despite there being no difference in fever, during-pandemic patients had an increased prevalence of sepsis (12.5 vs. 39.3%, *p*-value < 0.001) and pneumonia (68.8 vs. 41.8%, *p*-value < 0.001), but fewer positive bacterial culture results (87.5 vs. 10.8%, *p*-value < 0.001) (Table 1).

### 3.2. Pathogenic Bacteria Between Groups

Blood and sputum were the most usual specimens of the before- and during-pandemic patients, respectively (Table 2). Most of the pathogenic bacteria in both groups were Gram-negative (60.2%). The three most common Gram-negative bacteria were *Klebsiella pneumoniae* (32.1%), *Acinetobacter baumannii* (21.4%), and *Escherichia coli* (16.1%). There were differences in Gram-positive bacterial dominance between groups. *Streptococcus viridans*, *Staphylococcus aureus*, and *Staphylococcus epidermidis* were the three most prevailing Gram-positive bacteria in before-pandemic patients, whereas *S. aureus*, *S. epidermidis*, and *S. haemolyticus* were the three most ecumenical Gram-positive bacteria in during-pandemic patients.

The bacteria found in the before-pandemic group were not as varied as those found in the during-pandemic group. There were Gram-negative bacteria that were only found during the COVID-19 pandemic, for example, *Burkholderia cepacia*, *Enterococcus faecalis*, *Enterococcus faecium*, extended-spectrum beta-lactamase (ESBL)-producing *E. coli*, *Granulicatella adiacens*, ESBL-producing *K. pneumoniae*, *Micrococcus luteus*, *Pseudomonas aeruginosa*, *Serratia marcescens*, *Sphingomonas paucimobilis*, and *Stenotrophomonas maltophilia*; there were Gram-positive bacteria such as methicillin-resistant *S. aureus* (MRSA), *S. capitis*, *S. hominis*, and *S. pyogenes* (Table 3).

### 3.3. Risk Factors of Bacterial Infection After AIS Between Groups

According to the significance level of a *p*-value < 0.25 in the univariate analysis of the risk factors for bacterial infection after AIS before and during the COVID-19 pandemic, intensive care stay, fever, sepsis, pneumonia, COVID-19, multiple infarct lesions, the use of a ventilator, a urinary catheter, antibiotics, steroids, TPN, and transfusion, and procedures such as DSA were included in the multivariate logistic regression analysis. Fever, pneumonia, multiple infarct lesions, and the use of ventilators, urinary catheters, antibiotics, transfusion, and DSA were indicated as confounding variables.

The variables associated with a risk of bacterial infection during the COVID-19 pandemic with a *p*-value < 0.05 were intensive care stay (OR 0.22; 95%CI 0.13–0.39, *p*-value < 0.001), sepsis (OR 1.99; 95%CI 1.12–3.53, *p*-value = 0.019), COVID-19 infection (OR 4.81; 95%CI 2.64–8.76, *p*-value < 0.001), the use of steroids (OR 0.31; 95%CI 0.14–0.67, *p*-value = 0.003), and the use of TPN (OR 0.34; 95%CI 0.13–0.86, *p*-value = 0.022). The risk factors of bacterial infection are listed in Table 4.

## 4. Discussion

There were more male patients in this study, which is in line with previous studies [31,32,33]. The median age of the participants, 60 (14.0) years, is lower than in previous studies [8,34]. Age and sex did not significantly differ from one another, suggesting that they had no bearing on the cases of infection after AIS.

### 4.1. Prevalence of Bacterial Infection After Acute Ischemic Stroke (AIS)

The prevalence of bacterial infection after AIS based on the positive culture in our study was 17.9%. Even if we only included acute ischemic stroke patients within seven days, this result is consistent with the range of prevalence in previous studies, which was 5–65% [31,32,33,34,35]. However, the prevalence of bacterial infection during the COVID-19 pandemic (10.8%) was lower than before the COVID-19 pandemic (87.5%). During the COVID-19 pandemic, all patients underwent microbiological culture to discover whether they had a secondary infection or only had COVID-19 because the most common symptom of COVID-19 was fever, which can be caused by bacterial or viral infections. To rule out bacterial causes of infectious diseases, identify the etiology, and direct patient management, those patients underwent microbiological culture [36]. Therefore, despite a large number of febrile patients being cultured, the positivity rate of the culture was lower as most of the infections were due to viral infections of COVID-19. On the contrary, before the COVID-19 pandemic, patients who underwent microbiological culture were only patients with signs or symptoms due to infection; besides, microbiological culture is the gold standard for the diagnosis of infection.

Whereas pneumonia was the most prevalent clinical manifestation of infections of patients in both groups who underwent microbiological culture, followed by sepsis and fever, the sputum was not the most usual specimen for microbiological culture during the COVID-19 pandemic. Blood specimens were the most common, probably due to many patients showing both fever and sepsis signs and symptoms.

Among Gram-positive and Gram-negative microorganisms in this study, *Staphylococcus* sp. and *Klebsiella* sp. were most frequently observed, respectively. *S. aureus* and Gram-negative bacteria are known to cause pneumonia due to the aspiration of endogenous material from the colonized oropharynx. These pathogens typically cause nosocomial infections that occur in hospitals. *Streptococcus* sp. remains the most detected pathogen in cases of community-acquired pneumonia [35].

### 4.2. Risk Factors of Bacterial Infection After Acute Ischemic Stroke (AIS)

There were statistically significant differences based on the univariate analysis in intensive care stay, sepsis, pneumonia, COVID-19 infection, multiple lesions of infarct, the use of a ventilator, an NGT, antibiotics, and steroids, TPN, tracheostomy, DSA procedures, and the bacterial growing culture between the two groups (*p*-value < 0.05). However, the risk factors of bacterial infection after AIS after existing confounding factors were eliminated were intensive care stay, sepsis, COVID-19 infection, the use of steroids, and TPN.

Intensive care stay decreased the risk of bacterial infection after AIS during the COVID-19 pandemic by 22.4%. Stroke patients admitted to intensive care units often have more severe strokes. During the COVID-19 pandemic, data from previous studies suggest that underlying cerebrovascular diseases were associated with poor COVID-19 outcomes. In spite of pneumonia being a confounding variable in this study, pneumonia at admission is indicative of moderate or severe COVID-19 [37]. Patients with severe COVID-19 should be closely monitored and it indicates the need for intensive care since rapid progression from moderate to severe acute respiratory distress syndrome may occur [38]. Prioritizing patients with COVID-19 for care in a professional medical facility, especially in intensive care, may help reduce the mortality rate in the COVID-19 epidemic [39]. A number of clinical trials that studied the effects of prophylactic antibiotics in AIS patients showed that the use of prophylactic antibiotics could reduce the incidence of these infections, notwithstanding that there were no long-term advantages to antibiotics in terms of neurological outcomes, mortality, or morbidity [40].

COVID-19 increased the risk of bacterial infection after AIS 4.81 times. A number of previous studies showed that some COVID-19 patients were predisposed to bacterial infections. Critical patients with COVID-19, prior antibiotic use, and invasive therapy were identified as the three risk factors of overall bacterial infections. Invasive therapies, including ventilators, blood devices, and urinary catheters, were often used in critical patients with COVID-19. Retrospective studies have shown that in COVID-19 patients, especially those who were admitted into the ICU, a longer duration of ventilation, blood devices, and urinary catheters were associated with higher bacterial superinfection rates [41].

Sepsis in COVID-19 increased the risk of bacterial infection after AIS 1.93 times, though there was a lower prevalence of bacterial infection compared to before the COVID-19 pandemic. In a previous study, no bacteria were isolated in up to 42% of sepsis cases, suggesting that sepsis may be of a nonbacterial etiology, notwithstanding that bacteria have been shown to be the predominant pathogens of sepsis caused by infection, while the reported proportions of viruses were meager. Severe infections associated with COVID-19 have sparked a debate or at least raised awareness about viral sepsis, and the viral etiology has become more accepted as an essential cause of sepsis. Besides the diagnosis of sepsis using sepsis criteria, diagnosing viral sepsis depends on identifying the cause of the sepsis as a respiratory virus [42]. As many COVID-19 patients meet the diagnostic criteria for sepsis, Li H et al. [43] hypothesized that the process known as viral sepsis is a crucial step in the mechanism of COVID-19. In this study, blood specimens were examined by PCR to diagnose COVID-19. Therefore, there was a possibility that sepsis in our study was coronavirus-related sepsis; so, the prevalence of bacterial infection was lower compared to before the COVID-19 pandemic.

Corticosteroids have been shown in vivo to reduce inflammation associated with a dysregulated immune response, potentially reducing mortality if given early in severe COVID-19 cases. In the setting of severe COVID-19, where the initial immune response has not cleared the infection and has entered the pulmonary phase, the proposed benefit of introducing corticosteroids is thought to be due to the downregulation of immune-mediated lung injury and cytokine storm [44]. Systemic steroids, when used appropriately, modulate the immune response and have been shown to improve survival in COVID-19 patients, especially helping reverse septic shock in bacterial sepsis [45].

Most stroke patients have varying degrees of functional impairment, and rehabilitation is essential for patients to achieve functional recovery [46]. Following a stroke, dysphagia is highly prevalent [47]. Stroke patients who experience dysphagia or a decreased level of consciousness are often treated with an NGT to maintain each patient’s nutrition and hydration and avoid the risk of choking or aspiration, which are predisposing factors for pneumonia [48]. Using TPN in this study reduced the risk of bacterial infection after AIS during the COVID-19 pandemic by 33.8%. Patients should start receiving tubes within 24 h of being admitted to the hospital since early tube feeding is linked to better survival following stroke as a result of COVID-19 in hospitalized patients with a persistent pro-inflammatory state and recurring gastrointestinal complaints, which are associated with poor nutritional status [49,50]. A significant prevalence of dysphagia stands as the primary determinant of malnutrition in stroke patients due to inadequate meal intake [51]. Malnutrition is directly related to poor immune response and clinical evolution, which are associated with poor prognosis, increased mortality, and deteriorated outcomes in patients with stroke.

Research using an ischemic stroke mouse model revealed no discernible correlation between the risk of infection and the site of the infarct. The degree of cerebral ischemia rather than the location of the cerebral infarct is more critical in determining the risk of AIS. This suggests that increased susceptibility to infection in mice with larger cerebral infarcts was associated with a remarkable systemic change in immune cell populations, as demonstrated by a profound decrease in the lymphocyte–neutrophil ratio in the blood and lungs of post-stroke animals. It was proposed that infarct volume is of greater importance than infarct location in the risk of post-stroke immune alterations and infection [52]. A study on factors that predict immunologic alterations and subsequent infections in AIS revealed that stroke-induced immunodepression syndrome was swayed by infarct volume [13]. In this study, we did not specify where the infarct location was but differentiated the infarct into common single or multiple categories, and there were a lack of data on infarct volume. These might result in multiple infarct lesions, confounding the risk of bacterial infection after AIS.

Even though we included all of the complete medical records that accompanied the microbiological culture data, this study has several limitations. This study was conducted retrospectively, so for some key baseline variables, the data were incomplete or unavailable, resulting in less reliability in a prospective study. We could not report some of the risk factors, for instance, smoking status, stroke severity, stroke etiology, specific infarct location, infarct volume, and other comorbidities or treatments due to a lack of resources, which were limiting factors in the analysis. This was a single-center study, and the sample size of patients before the COVID-19 pandemic was relatively small, so there might be the possibility that this would affect the results, rendering them not applicable in a different setting.

## 5. Conclusions

From this study, the following conclusions may be made. First, the bacterial infection rate in acute ischemic stroke patients based on culture, the gold standard, was 17.9%, which is within the range of previous studies. However, this infection rate was influenced by the characteristics and clinical conditions of the patients, indicated by differences in the prevalence of bacterial infection after AIS, which declined following the start of the COVID-19 pandemic. Second, *Staphylococcus aureus* and Gram-negative bacteria, known to cause pneumonia due to aspiration, predominate among bacteria that cause infection. Third, whilst there are significant differences in pneumonia, multiple lesions of infarct, the use of a ventilator, an NGT, and antibiotics, tracheostomy, and DSA procedures between patients before and after the COVID-19 pandemic, these factors confound the risk factors of bacterial infection after AIS, which were intensive care stay, sepsis, COVID-19 infection, the use of steroids, and TPN.

## Figures and Tables

**Table 1:** Subject characteristics.

	Total		Before Pandemic		During Pandemic		*p*-Value
	*n*	%	*n*	%	*n*	%	
Subject	519	100	48	9.2	471	90.8	-
Age, mean (SD)	60.7 (10.5)		59.0 (11.3)		60.9 (10.4)		-
Age group, years, *n* (%)							
<60	273	52.6	22	45.8	251	53.3	0.324
≥60	246	47.4	26	54.2	220	46.7	
Sex, *n* (%)							
Male	347	66.9	35	72.9	312	66.2	0.349
Female	172	33.1	13	27.1	159	33.8	
Care unit, *n* (%)							
Intensive	163	31.4	31	64.6	132	28.0	<0.001 *
Non-intensive	356	68.6	17	35.4	339	72.0	
Clinical manifestation of infection, *n* (%)							
Fever	117	22.5	6	12.5	111	23.6	0.080
Sepsis	191	36.8	6	12.5	185	39.3	<0.001 *
Meningitis/encephalitis	17	3.3	3	6.3	14	3.0	0.743
Pneumonia	230	44.3	33	68.8	197	41.8	<0.001 *
Infected wound	8	1.5	1	2.1	7	1.5	0.368
UTI	5	1.0	1	2.1	4	0.8	0.386
Comorbidity, *n* (%)							
DM	156	30.1	17	35.4	139	29.5	0.395
HIV	20	3.9	1	2.1	19	4.0	1.000
COVID-19	307	59.2	-	-	307	65.2	<0.001 *
Leukocyte/μL, *n* (%)							
>10,000	325	64.6	29	63.0	296	64.8	0.815
5000–10,000	178	35.4	17	37.0	161	35.2	-
<5000	16	8.2	2	10.5	14	8.0	0.660
History of infarct location, *n* (%)							
Single lesion	111	21.4	12	25.0	99	21.0	0.522
Multiple lesions	319	61.5	21	43.8	298	63.3	0.008 *
Medical device, *n* (%)							
Ventilator	52	10.0	11	22.9	41	8.7	0.005 *
CVC	124	23.9	12	25.0	112	23.8	0.850
NGT	39	7.5	7	14.6	32	6.8	0.076
Urinary catheter	19	3.7	-	-	19	4.0	0.241
Treatment, *n* (%)							
Thrombolysis	-	-	-	-	-	-	-
Thrombectomy	-	-	-	-	-	-	-
Antibiotic	341	65.7	40	83.3	301	63.9	0.007 *
Steroid	51	9.8	17	35.4	34	7.2	<0.001 *
TPN	33	6.4	11	22.9	22	4.7	<0.001 *
Transfusion	25	4.8	5	10.4	20	4.2	0.070
Procedure, *n* (%)							
Tracheostomy	24	4.6	4	8.3	20	4.2	0.265
DSA	9	1.7	6	12.5	3	0.6	<0.001 *
Head surgery	5	1.0	-	-	5	1.1	1.000
Microbiological culture result, *n* (%)							
Positive bacterial	93	17.9	42	87.5	51	10.8	<0.001 *
Negative bacterial	426	82.1	6	12.5	420	89.2	

* *p*-value < 0.05 indicates statistically significant related variables. COVID: coronavirus disease; CVC: central venous catheter; DM: diabetes mellitus; DSA: digital subtraction angiography; HIV: human immunodeficiency virus; μL: microliter; NGT: nasogastric tube; SD: standard deviation; TPN: total parenteral nutrition; UTI: urinary tract infection.

**Table 2:** Positivity rate of specimens.

	Total		Before Pandemic						During Pandemic					
			Total		Positive		Negative		Total		Positive		Negative	
	*n*	%	*n*	%	*n*	%	*n*	%	*n*	%	*n*	%	*n*	%
CSF	17	3.3	3	6.3	1	2.4	2	33.3	14	3.0	1	2.0	13	3.1
Blood	401	77.3	2	4.2	-	-	2	33.3	399	84.7	10	19.6	389	92.6
Bronchial lavage	1	0.2	-	-	-	-	-	-	1	0.2	-	-	1	0.2
Sputum	86	16.6	41	85.4	40	95.2	1	46.7	45	9.6	36	70.6	9	2.1
Pleural fluid	1	0.2	-	-	-	-	-	-	1	0.2	-	-	1	0.2
Ulcer	8	1.5	1	2.1	1	2.4	-	-	7	1.5	3	5.9	4	1.0
Urine	5	1.0	1	2.1	-	-	1	16.7	4	0.8	1	2.0	3	0.7

CSF: cerebrospinal fluid.

**Table 3:** Distribution of bacteria.

	Total		Before Pandemic		During Pandemic	
	*n*	%	*n*	%	*n*	%
**Gram-negative bacteria**	56	60.2	23	41.7	33	58.9
*Acinetobacter baumannii*	12	21.4	7	30.4	5	15.2
*Burkholderia cepacia*	1	1.8	-	-	1	3.0
*Enterobacter cloacae*	3	5.4	1	4.3	2	6.1
*Enterococcus faecalis*	1	1.8	-	-	1	3.0
*Enterococcus faecium*	1	1.8	-	-	1	3.0
*Escherichia coli*	6	10.7	3	13.0	3	9.1
*Escherichia coli* (ESBL)	3	5.4	-	-	3	9.1
*Granulicatella adiacens*	1	1.8	-	-	1	3.0
*Klebsiella oxytoca*	3	5.4	3	13.0	-	-
*Klebsiella pneumoniae*	16	28.6	9	39.1	7	21.2
*Klebsiella pneumoniae* (ESBL)	2	3.6	-	-	2	6.1
*Micrococcus luteus*	1	1.8	-	-	1	3.0
*Pseudomonas aeruginosa*	3	5.4	-	-	3	9.1
*Serratia marcescens*	1	1.8	-	-	1	3.0
*Sphingomonas paucimobilis*	1	1.8	-	-	1	3.0
*Stenotrophomonas maltophilia*	1	1.8	-	-	1	3.0
**Gram-positive bacteria**	37	39.8	19	51.4	18	48.6
*Staphylococcus aureus*	9	1.7	2	10.5	7	38.9
*Staphylococcus aureus* (MRSA)	1	0.2	-	-	1	5.6
*Staphylococcus capitis*	1	0.2	-	-	1	5.6
*Staphylococcus epidermidis*	6	1.2	4	21.1	2	11.1
*Staphylococcus epidermidis* (MRSE)	3	0.6	1	5.3	2	11.1
*Staphylococcus haemolyticus*	2	0.4	-	-	2	11.1
*Staphylococcus hominis*	1	0.2	-	-	1	5.6
*Streptococcus agalactiae*	2	0.4	1	5.3	1	5.6
*Streptococcus pyogenes*	1	0.2	-	-	1	5.6
*Streptococcus viridans*	11	2.1	11	57.9	-	-

ESBL: extended-spectrum beta-lactamase, MRSA: methicillin-resistant *Staphylococcus aureus*, MRSE: methicillin-resistant *Staphylococcus epidermidis.*

**Table 4:** Multivariate analysis of factors associated with infection after acute ischemic stroke.

Variable	Initial Model			Final Model		
	OR	95%CI	*p*-Value	OR	95%CI	*p*-Value
Intensive care	0.22	0.12–0.41	<0.001 *	0.22	0.13–0.39	<0.001 *
Fever	2.05	0.93–4.50	0.07	-	-	-
Sepsis	1.66	0.92–3.01	0.09	1.99	1.12–3.53	0.019 *
Pneumonia	1.06	0.57–1.94	0.86	-	-	-
COVID-19	4.89	2.63–9.12	<0.001 *	4.81	2.64–8.76	<0.001 *
Multiple lesions of infarct	0.99	0.99–1.00	0.25	-	-	-
Ventilator	0.97	0.38–2.50	0.957	-	-	-
Urinary catheter	0.57	0.16–1.98	0.375	-	-	-
Antibiotic	0.73	0.39–1.35	0.314	-	-	-
Steroid	0.33	0.14–0.75	0.009 *	0.31	0.14–0.67	0.003 *
TPN	0.32	0.10–0.96	0.043 *	0.34	0.13–0.86	0.022 *
Transfusion	1.47	0.48–4.55	0.503	-	-	-
DSA	0.22	0.04–1.19	0.079	-	-	-

* *p*-value < 0.05 indicates statistically significant related variables. CI: confidence interval; COVID: coronavirus disease; DSA: digital subtraction angiography; OR: odds ratio; TPN: total parenteral nutrition.

## Data Availability

Researchers can access the data upon reasonable request or by contacting the corresponding author personally.
